# Systemic schistosomiasis and large bowel perforation: An unexpected surgical urgency. Report of a case and literature review

**DOI:** 10.1002/ccr3.2138

**Published:** 2019-04-05

**Authors:** Antonino Agrusa, Giuseppe Di Buono, Salvatore Buscemi, Ilaria Canfora, Brenda Randisi, Giulia Bonventre, Leonardo Gulotta, Elisa Maienza, Vincenzo Sorce, Giorgio Romano, Gaspare Gulotta

**Affiliations:** ^1^ Section of General and Urgent Surgery, Department of Surgical, Oncological and Oral Sciences University of Palermo Palermo Italy

**Keywords:** colonic perforation, emergency surgery, systemic schistosomiasis

## Abstract

In the presence of suggestive clinical picture (high eosinophil count and multiple CT scan granuloma‐like lesions), schistosomiasis should be taken into account in case of suspected bowel perforation even if common risk factors are not identified through anamnesis.

## 1 INTRODUCTION

Schistosomiasis is a multifactorial chronic infection due to a trematode of the genus Schistosome. World Health Organization (WHO) still considers schistosomiasis as second only to malaria in socioeconomic importance worldwide.^1^ It is one of the neglected tropical diseases (NTDs), a group of 17 or more chronic parasitic diseases and related infections that represent the most common illnesses of the world's poorest people. The distribution of the NTDs is prevalent in the South America, Europe, sub‐Saharan Africa, China and East Asia, India and South Asia, Central Asia, in the Middle East and North Africa (MENA).^2^ In these areas, schistosomiasis is known as an endemic disease; on the contrary, in occidental countries it is considered very rare thanks to the better living standards, advanced hygiene conditions, lower poverty rate, and nonpredisposing geographical factors. Some authors have drawn attention to underreporting of the ectopic forms of the disease, which would be directly associated with the difficulty in diagnosis and with the lack of proper investigation for the confirmation of such diagnostic hypotheses.

## 2 PRESENTATION OF THE CASE

**Figure 1:**
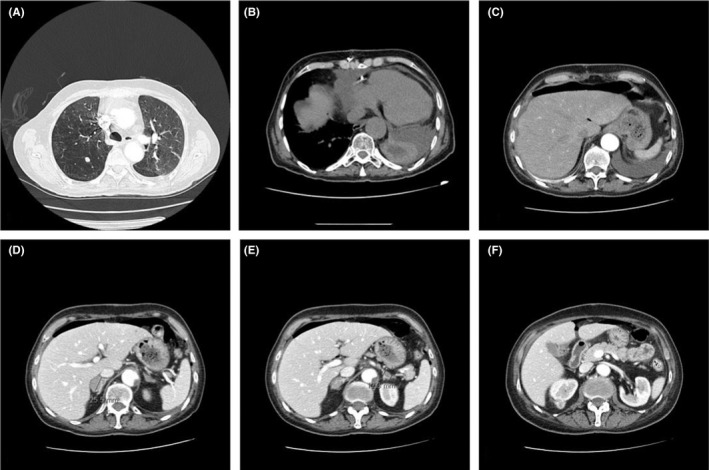
CT scan: (A) calcific nodule in the right lung; (B) significant pericardial effusion; (C) free peritoneal air; (D, E) an adenoma‐like lesion in the right adrenal gland and a similar one in the left adrenal gland; and (F) exophytic lesion of the upper pole of the right kidney

We report the clinical case of a 63‐year‐old Caucasian Italian housewife, who came to our observation from the emergency room complaining an acute progressive abdominal pain and distension, dyspepsia, nausea, diarrhea, and in the last 2 days rectal passage of blood and mucous. She also referred to recurring palpitation and weakness since 3 months. She presented a severe grade of anemia (Hb 7.4 g/dL) requiring blood transfusion, piastrinopenia, a significantly high level of LDH (554 U/L), and leukocytosis with hypereosinophilia. Blood pressure was 95/60 mm Hg, heart rate 95/min, and Glasgow Coma Scale 15. She had an history of atherosclerotic disease and an acute myocardial infarction with cardiac arrest, undergone double coronary bypass. After that event, a thoracic aortic dissection occurred, so an endovascular prosthesis was positioned. During the long recovery because of an acute cardiac tamponade, she underwent surgery again and a pericardial‐pleural window was made. After 2 weeks, she developed apical right pneumothorax, due to emphysematous bullae rupture, and pneumomediastinum was treated by drainage insertion. On our clinical inspection, the patient presented many millimetric papular lesions and petechiae on the anterior part of the thorax and on the central part of the abdomen. Other similar centimetric lesions could be detected on the anterior surface of both legs and arms. She regularly took anticoagulant and cardioaspirin since the first cardiac surgery. To physical examination, the abdomen was no tractable, with signs of peritonitis. Shallow breathing was evident. Abdominal CT scan revealed free subphrenic air and free fluid, due to a probable perforation of the upper digestive tract, with thickening and edema of the intestinal loops. No air‐fluid levels were seen. Other incidental findings were as follows: two calcific nodules in the right lung (10 and 5 mm diameter max); significant pleural and pericardial effusion; an adenoma‐like lesion in the right adrenal gland (25 mm diameter max) and a similar one in the left adrenal gland (12 mm diameter max); and a small calcific left ovarian cyst. CT scan showed also an exophytic lesion of unclear nature (22 mm diameter max) in the upper pole of the right kidney (Figure 1). Small eccentric thrombotic clots were detected in the suprarenal aorta lumen, without hemodynamic meaning. All these reports were unknown at the previous imaging reports introduced by the patient (chest X‐ray and CT scan; abdomen CT scan, performed about 3 months before). Because of the important pericardial effusion, in consideration of the clinical history a preoperative echocardiogram was needed to study the hemodynamic patient status. It helped us to exclude a cardiac tamponade but also revealed a pericardial effusion with a separation of pericardial layers of 1.5 cm and collapse of the vena cava, likely due to hypovolemia. No ventricular failure was detected. After this assessment of cardiac condition,^3^ she was sent to operating room. Despite the preoperative suspicion of gastric perforation, we did not perform a laparoscopic approach because of the high risk of the patient (ASA score 4) and severe grade of anemia and we decided for a midline laparotomy.^4^, ^5^, ^6^, ^7^, ^8^ At the opening of the abdominal cavity, air and copious quantities of feces came out, as a massive colic perforation and faecalis peritonitis. We detected multiple centimetric perforation areas on the medium transverse colon wall with many millimetric ischemic areas along the entire length of the right colon. Both the mucosa and the sierosa were congested, with a brownish red appearance. All these macroscopic data could lead to an ischemic origin of the perforation. We detected no palpable macroscopic lesions during the exploration, besides the CT scan‐evidenced left ovarian one. Left colon was normal without evidence of stenosis or inflammation. We carried out an extended right colectomy but no ileo‐cholic anastomosis could be done in consideration of the severe sepsis and the associated comorbidities, and we performed a terminal ileostomy. We washed the peritoneal cavity with more than 10 liters of warm saline solution, and we used a direct closure of the abdomen without advanced dressing (eg, Negative Wound Pressure Therapy, NWPT, on open abdomen). After surgical treatment, the patient went to intensive care unit (ICU) for management of septic shock and she received antibiotic therapy with piperacillin + tazobactam, metronidazole, and meropenem. CT scan of the chest and abdomen performed 7 days after surgery showed a bronchopneumonic process with no significant abdominal findings. We transferred the patient to internal medicine unit but she died of heart attack 4 weeks after surgery. Pathological examination revealed an irregular mucosa with ulcerated areas and aphtoid lesions. Histology showed multiple areas of erosions and thinning altered with necrosis zones ending in perforation. Several spherical calcifications were objectivated, mostly in the subserosa and the submucosa, suggestive of calcificated Schistosoma larvae's eggs in advanced involution phase.

## 3 DISCUSSION

Because of the rarity of this infection in the industrialized countries, considering patient's anamnesis (she referred to be a housewife with no recent travels in endemic regions) and complex clinical picture, this finding is worthy of an accurate analysis. Colorectal perforation may be due to neoplastic, inflammatory, or ischemic disease as well as traumatic injuries. Complicated diverticulitis and colorectal cancer are the most common causes.^9^, ^10^, ^11^ However, several benign etiologies should be taken into account except for trauma perforation (which include iatrogenic perforations, blunt abdominal trauma, and stab wounds)^9^, ^12^, ^13^, ^14^, ^15^ excluded by clinical history, Necrotic acute pancreatitis, ulcerative colitis, stenosis of colorectal anastomosis with obstruction and idiopathic large bowel perforation are rarely mentioned (5.1%).^9^, ^16^, ^17^ Hepatic and intestinal schistosomiasis is the most common form of the chronic disease and usually results from heavy *Schistosoma mansoni* infection.^18^, ^19^ Egg‐laying worms are present in the intestinal microvessels especially in the distribution of the inferior mesenteric venous plexus. In the large intestine, Schistosoma eggs are mainly distributed in the loose submucosa and in the subserosa layer where infrequently make multiple granulomas. Subsequently, the infection involves the muscularis mucosa and the overlying mucosa is either denuded forming small superficial ulcers and hyperplastic changes. Sandy patches develop when the submucosa becomes thickened by fibrous tissue containing several calcified eggs; the overlying mucosa becomes atrophic and acquires a granular dirty yellowish appearance.^20^ Colonic mucosa of affected patient is usually edematous and congested with petechial hemorrhage in acute schistosomal colitis cases.^21^ Ulceration is common; the ulcerated areas appear blackish gray in color caused by superficial hemorrhage and are frequently secondarily infected. At this point, intestinal schistosomiasis can be diagnosed by finding eggs in tissue specimens from rectal and intestinal biopsies.^22^ According to our macroscopic and microscopic report, we can attribute the perforation to parasitic large bowel infection. Ectopic schistosomiasis occurs when the parasite eggs or adult forms are located far from the portal system. Ectopic eggs deposition can lead to additional clinical syndromes, including involvement of skin, lungs, brain, muscles, adrenal glands, genitalia, and eyes. In this report, thanks to the CT‐images, we can support the hypothesis of a systemic case of chronic schistosomiasis. CT scan of the thorax detected two calcific nodules as unspecified opacities in the upper lobe of the right lung (10 and 5 mm diameter max). No history of dyspnea was referred with no significant findings at physical examination. Pulmonary schistosomiasis, although rare, can be divided into two categories: acute and chronic. Chronic and recurrent infection develops in persons living or traveling in endemic areas.^23^ The common signs at the chest CT scan in pulmonary schistosomiasis are multiple small pulmonary nodules ranging from 2 to 15 mm with ground glass opacity halo such as in our case report.^24^ This patient did not report gynecological history. Abdominal CT scan identified a small left ovarian cyst, with partially defined borders, calcific walls, and internal fluid. According to the data in literature, the ovaries are the most affected organs in case of female genital schistosomiasis via the rectovaginal septum blood flow.^25^, ^26^ The typical radiological presentation consists in calcific aspect of the mass due to the presence of schistosomal granulomas surrounding eggs of *S mansoni* with sovrafluid content forming by inflammatory response.^28^ These signs are expected in cases of chronic genital schistosomiasis infection and were detected in our patient. Despite the age of the patient (63 years), we did not perform the left ovariectomy because the emergency surgical management was aimed to treat the acute condition and we thought to make the ovariectomy at the same time of recanalization. Abdominal CT scan showed adenoma‐like lesions in the right and left adrenal gland.^29^, ^30^ We did not find any previously reported cases of adrenal ectopic schistosomiasis but we can mention a single case report of an adrenal schistosomiasis incidentally diagnosed in association with adenoma.^36^ In our case report, we do not have an histopatological examination of the adrenal gland because there is no indication to perform an adrenal biopsy or adrenalectomy in urgent setting with no specific diagnosis, but the association with the peculiar clinical presentation, the histological diagnosis of schistosomiasis and the CT scan findings can support the suspect of systemic schistosomiasis with probable adrenal involvement. In this case report, we treated an acute event, intestinal perforation, caused by systemic schistosomiasis. The preoperative suspicion, however, would not have changed our surgical management due to severe septic shock.

## 4 CONCLUSION

Schistosomiasis is very rare in occidental countries; meanwhile, in chronic form it is still considered as the second only to malaria in socioeconomic importance in endemic region. In this case, we present a woman with no risk factors, negative professional exposure, or recent travel history, who was living in an urban area. According to our analysis, many districts were involved by the infection, supporting the thesis of a systemic schistosomiasis even though the hypothesis of an ischemic origin of the bowel perforation was the most likely scenario. In the presence of suggestive clinical picture (high eosinophil count and multiple CT scan granuloma‐like lesions), schistosomiasis should be taken into account in case of suspected bowel perforation even if common risk factors are not identified through anamnesis.

## CONFLICT OF INTEREST

None declared.

## AUTHOR CONTRIBUTION

AA: involved in study design, collected the data, analyzed and wrote the data. GDB: involved in study design, collected the data, analyzed and wrote the data. SB: involved in study design, collected the data, analyzed and wrote the data. IC: involved in study design, collected the data, analyzed and wrote the data. BR: collected the data. GB: collected the data. LG: collected the data. EM: collected the data. VS: collected the data. GR: involved in study design, collected the data, analyzed and wrote the data. GG: involved in study design.
